# Ki-67 and MCM-2 in Dental Follicle and Odontogenic Cysts: The Effects of Inflammation on Proliferative Markers

**DOI:** 10.1100/2012/946060

**Published:** 2012-06-18

**Authors:** Nurhan Güler, Nil Çomunoğlu, Fatih Cabbar

**Affiliations:** ^1^Department of Oral and Maxillofacial Surgery, Faculty of Dentistry, Yeditepe University, No. 238 Bagdat Cd, 34728 Goztepe, Istanbul, Turkey; ^2^Pathology Service, Haydarpaşa Hospital, 34668 Uskudar, Istanbul, Turkey; ^3^Department of Pathology, Faculty of Medicine, Yeditepe University, 34755 Istanbul, Turkey

## Abstract

The aim of this study was to investigate whether there is any association between inflammation and the expression of markers of cell cycle entry (Ki-67 and MCM-2) in dental follicle (DF) of asymptomatic impacted teeth and odontogenic cysts. The study consisted of 70 DFs and 20 odontogenic cysts (radicular cyst (RC), dentigerous cyst (DC) and keratocytic odontogenic tumor (KCOT) located at posterior mandibular region. Histological findings of inflammation for all specimen and mucous cell prosoplasia, squamous metaplasia, glandular epithelium for all DFs were stained with hematoxyline and eosin, periodic acid schiff, alcian blue, and mucin. Epithelial cell proliferation was determined by using immunohistochemical labeling for Ki-67 and MCM-2. The histologic examinations showed 16% mucous cell prosoplasia, 54% squamous metaplasia, 20% glandular epithelium, 37% inflammation. Inflammation was detected in all RCs and %62 in DF, %43 in DC and KCOT. Positive correlation was found between the inflammation of DF and odontogenic cysts (*P* < 0.01). The mean Ki-67 and MCM-2 expressions were found 9, 64 ± 5, 99 and 6, 34 ± 3, 81 in DF, 11, 85 ± 9, 01 and 13, 6 ± 9, 94 in odontogenic cysts, respectively. While the mean Ki-67 expressions were statistically significant in DF and KCOT (*P* < 0.01), MCM-2 were significant in RC and KCOT (*P* < 0.01). MCM-2 expresion in RCs were statistically significant than KCOT (*P* < 0.01). The results of this study indicated that the higher MCM-2 expressions in RC than the KCOT might be related to the inflammation and this protein might be more sensitive to inflammation.

## 1. Introduction

 Odontogenic cysts, osteodestructive lesions affecting jaws, are arise from epithelial cells of dental follicle (DF) or from the remnants of odontogenic epithelium such as reduced enamel epithelium, malessez, hertwig epithelial shield, or rests of serres [1]. The most common cysts originated from these odontogenic cells are dentigerous cyst (DCs), keratocytic odontogenic tumor (KCOTs), and calcified odontogenic tumor [2, 3]. It is well known that the epithelial linings of both inflammatory and developmental cysts of odontogenic origin are primarily composed of squamous epithelium, but various forms of metaplasia and degeneration are observed in these epithelial linings such as mucous cells, ciliated cells, para and/or ortho-hyperkeratinization, and formation of hyaline bodies [4]. Radicular cyst (RC) is an odontogenic cyst of inflammatory stimulation of the epithelial rests of Malassez [5]. DC presents clinically as an aymptomatic unilocular radiolucency enclosing the crown of an unerupted or impacted tooth [3], and it can transform into more serious lesions, such as unicystic ameloblastoma [6]. Keratocystic odontogenic tumor (formerly odontogenic keratocysts, KOCT) is a unique cyst because of its locally aggressive behavior, high recurrence rate, and characteristic histological appearance [4]. RCs, DCs, and KCOTs show distinct growth patterns and biological behaviors. Increased activity of the epithelium, confirmed by previous study that has compared KCOTs with other odontogenic cysts, may explain the high recurrence rates of KCOTs [7].

Cell proliferation plays an important role in several biological and pathological events such as oral cysts and tumors [5, 7, 8]. The proliferative potential can be assessed by immunohistochemistry using monoclonal antibodies against specific cell cycle associated proteins. The proliferative activity of the epithelial lining of odontogenic cysts more particularly in KCOTs has been the subject of various investigations examining the expressions of p53, proliferating cell nuclear antigen (PCNA), Ki-67, as well as nucleolar organiser regions (AgNORs) [5, 7–10]. Among the immunohistochemical cell cycle markers, the monoclonal antibody Ki-67 has been the most widely used, which labels all active parts of the cell cycle, rises during the second half of the S phase, reaches a peak in the G2 and M phases, and rapidly degrades after mitosis with a half life of detectable antigen being an hour or less. It can be expressed actively in proliferating cells, in particular, neoplasms and its immunoreactivity, has been found to correlate closely with other variables of cell proliferation [7, 11].

Minichromosome maintenance proteins (MCM) expression can be used as a new marker in the determination of the proliferating cells. The main function of MCM proteins is cooperation with other factors in molecular mechanisms that form the replication fork and in regulation of DNA synthesis. MCM proteins form a ring-shaped complex, which is activated when other factors are bound. MCM 2–7 complex is one of the prereplication factors. Association of MCM 2–7 complex is a crucial moment initiating the replication fork. MCM proteins play a role in maintaining genome integrity and prevent rereplication once per cell cycle. Proliferating cells have high levels of MCM, whereas they are not detected in quiescent, differentiated or senescent cells. Recent results also indicate that the presence or absence of licensed origins has even deeper implications for cell biology, as it might play a role in determining the proliferative capacity of cells [12–14]. They are also potential useful markers of cell proliferation. This protein can be observed both in cells coming out of the cell cycle and during the cellular proliferation in the normal, premalignant, and neoplastic cells [14, 15]. Recent studies suggested that MCMs are good markers of proliferation activity degree, because they are highly expressed in a variety of tumors [12].

Under the influence of pathologic changes, however, dental follicles that possess reduced epithelium can proliferate into stratified squamous epithelium as far as originate dental cysts [16]. In this way, the microscopic analysis concomitant to immunohistochemical markers is relevant for understanding the behavior of DF in and odontogenic cysts. It appears that assessing the proliferative capacity of epithelial cells involved in odontogenic cysts may be useful in determining cyst progression/recurrence and presumably prognosis. Thus, the aim of this study was to investigate whether there is any association between inflammation and the expression of markers of cell cycle entry (Ki-67 and MCM-2) in dental follicle (DF) of asymptomatic impacted teeth and odontogenic cysts. 

## 2. Materials and Methods

 The study consisted of 70 DFs in 68 patients having at least 1 impacted mandibular third molar fully covered by mucosa, no history or sign of infection, or radiographically enlarged tissues surrounding impacted third molars and 20 inflammatory and developmental odontogenic cysts located at the posterior mandibular region (of 6 RCs, 7 DCs and 7 KCOTs) were included. Fifty females and 20 males (mean age 27 years, range 16–68 years) were included in DFs group and seven females and 13 males (mean age 39 years, range 22–67 years) were included in odontogenic cysts group. The study was approved by Yeditepe University clinical investigations ethic committee (2006) and informed consent was obtained from all participating patients. 

DFs of impacted teeth were carried out under local anesthesia by conventional third molar surgery and biopsy taken from odontogenic cyst. All specimens were placed immediately in 10% buffered formalin and processed to paraffin wax. Sections (5 *μ* thick) were cut from each block containing the DF and cysts specimens and stained with hematoxylin and eosin (H&E) for routine histologic examination. All slides were stained with periodic acid Schiff (PAS), Alcian blue, and mucin for the evaluation of mucous cell prosoplasia. Histologic appearance of normal DF described by Glosser and Campbell [16] was used and any soft tissue specimens with squamous epithelium spreading along the surface of the DF were considered as cystic [9]. Histologically, the epithelial components consisted of mucous cell prosoplasia, basal epithelial cells, squamous epithelium, and glandular epithelium, while mesenchymal components were inflammation, rests of the odontogenic epithelium, and calcified follicles.

Immunohistochemical method was described in our previous study [9]. Briefly, 5 *μ* thickness sections were prepared for incubations of Ki-67 (ScyTek, Logan, UT; 7 mL) and MCM-2 (MCM-2; CRCT2.1 [D1.9H5]; 1/100 dilution) antibodies according to manufacture's instructions. Dysplastic cervix epithelium was used as a positive control for Ki-67 and tonsillary tissue for MCM-2. Each slide was examined under a multihead light microscope and scored by pathologist. For each of the specimens, intensity and extent of Ki-67 and MCM-2 expression were evaluated by a comprehensive scoring formula. One hundred nuclei were assessed in each specimen and scored as a percentage of positively stained nuclei out of the total number of nuclei reprehensive microscopic fields counted. 

Histologic and immunohistochemical characteristics of epithelial and mesenchymal components of DF and odontogenic cysts were recorded and the Kruskal-Wallis test was used to compare data between groups. Two-tailed independent Student's *t* test was used to compare data between groups, whereas Mann-Whitney *U* test was used to make comparisons within groups. The level of significance was set at *P* < 0.05.

## 3. Results

**Figure 1:**
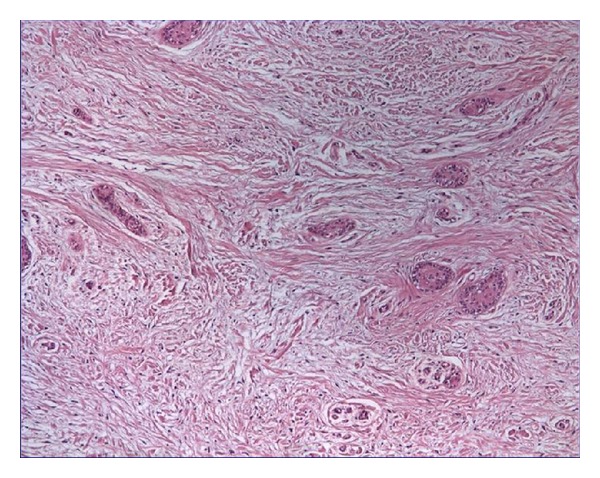
Rests of odontogenic epithelium (HE ×40).

**Figure 2:**
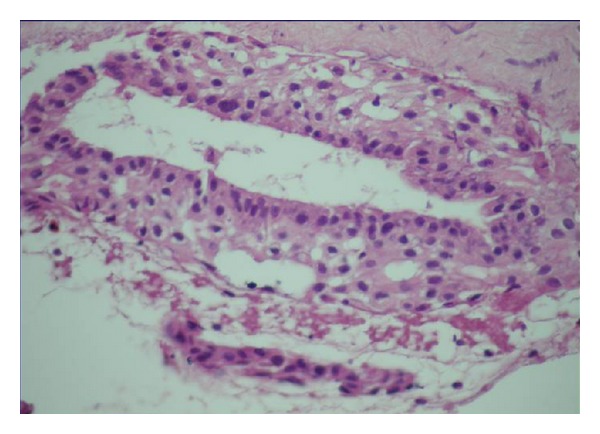
Odontogenic epithelium forming glandular structures (HE ×100).

**Figure 3:**
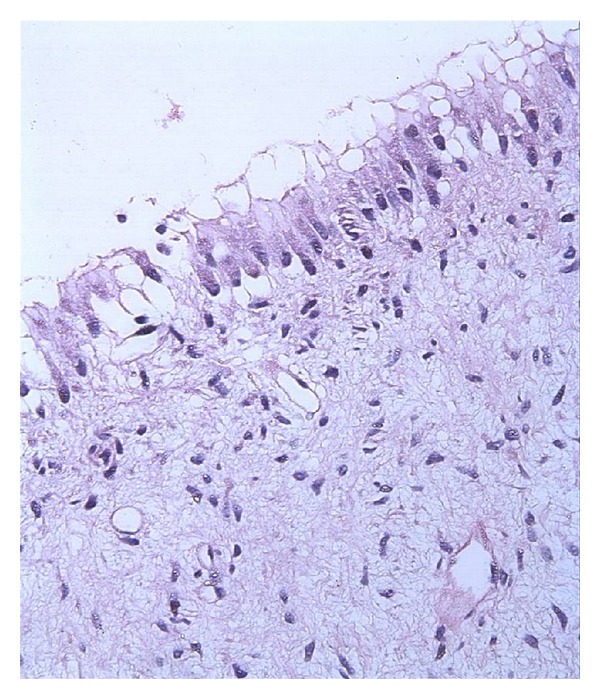
Mucous cell proplasia and fıbromyxoid type connective tissue (HE ×100).

**Figure 4:**
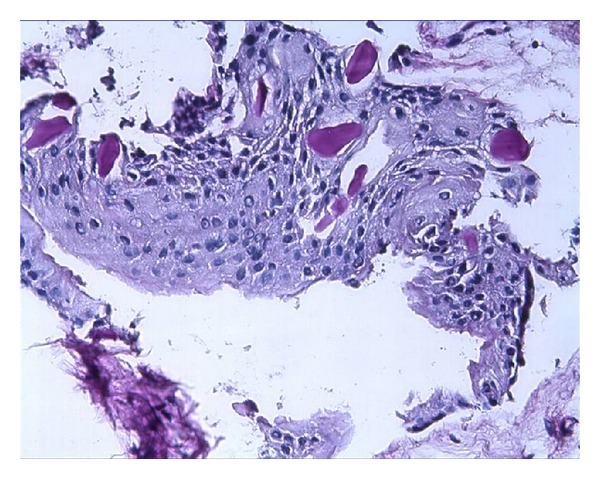
Squamous epithelium with pas positive globular formation-(PAS ×100).

**Figure 5:**
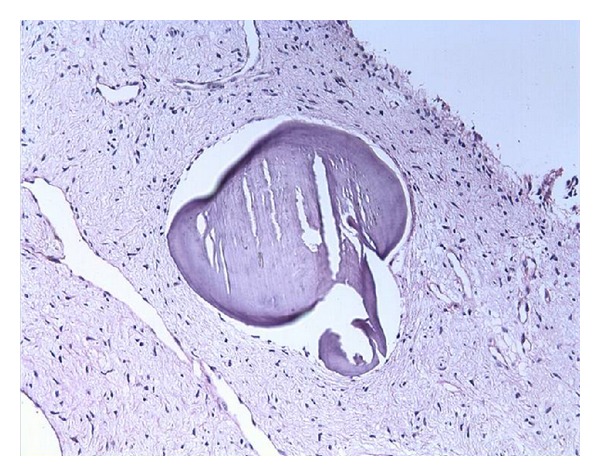
Calcification (PAS ×40).

The distribution of all specimens according to patient age, gender and localization is listed in Table 1. The patients were divided into age groups and of 42 (60%) DFs were found between 20 and 29, and 16 (22.85%) were 30 and older. The histologic examinations showed 16% mucous cell prosoplasia, 54% squamous metaplasia, 20% glandular epithelium, 37% inflammatory cells, and 44% rests of odontogenic epithelium in DF specimens (Figures 1, 2, 3, 4, and 5). The epithelium was detected in 53 (75,7%) and more frequently under 30 years old (77,4%) whereas 56,6% was detected between 20 to 29 age groups. A statistically significant relationship was found between the age and epithelium type of DF (*P* < 0.001).

Calcifications were seen in 42 (60%) DF and 2 (10%) odontogenic cysts (one DC and other in KCOT). Inflammatory changes were detected in all RCs while it was %62 in DF, %43 DCs and KCOTs. Positive correlation was found between the inflammation of DF and odontogenic cysts (*P* < 0.01). Inflammatory cells were seen in 47,4% of DFs which squamous epithelium was observed. 28,6% of DFs with calcifications also showed inflammatory cells.

**Figure 6:**
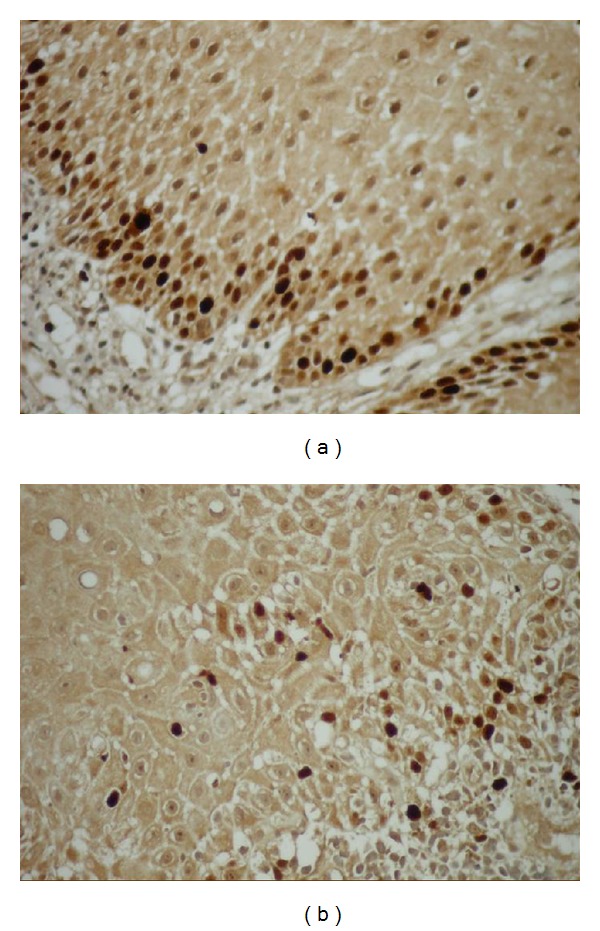
(a) Ki-67 expressions in the squamous epithelium (Ki-67 ×40). (b) MCM-2 expression in the squamous epithelium (MCM-2 ×40).

**Figure 7:**
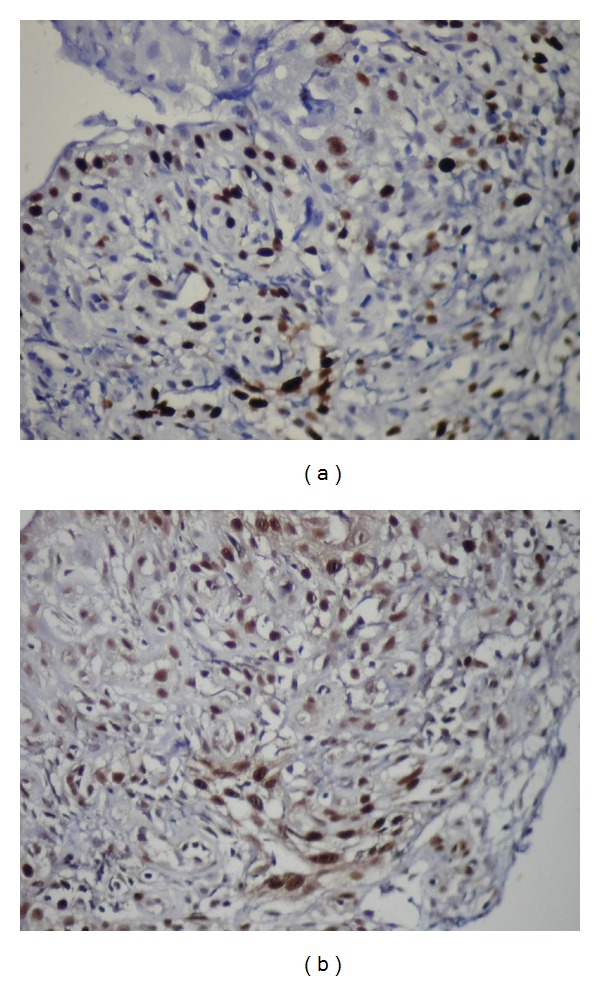
(a) The expressions of Ki-67 in lining epithelium of radicular cysts (Ki-67 ×40). (b) The expressions of MCM-2 in lining epithelium of radicular cysts (MCM-2 ×40).

**Figure 8:**
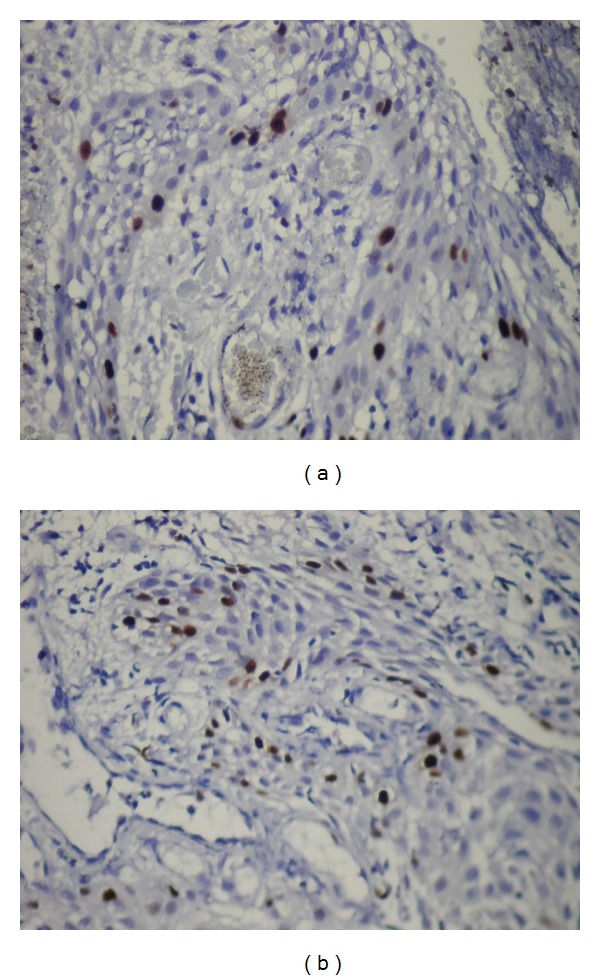
(a) The expressions of Ki-67 in epithelium of dentigerous cysts (Ki-67 ×40). (b) The expressions of MCM-2 in epithelium of dentigerous cysts (MCM-2 ×40).

**Figure 9:**
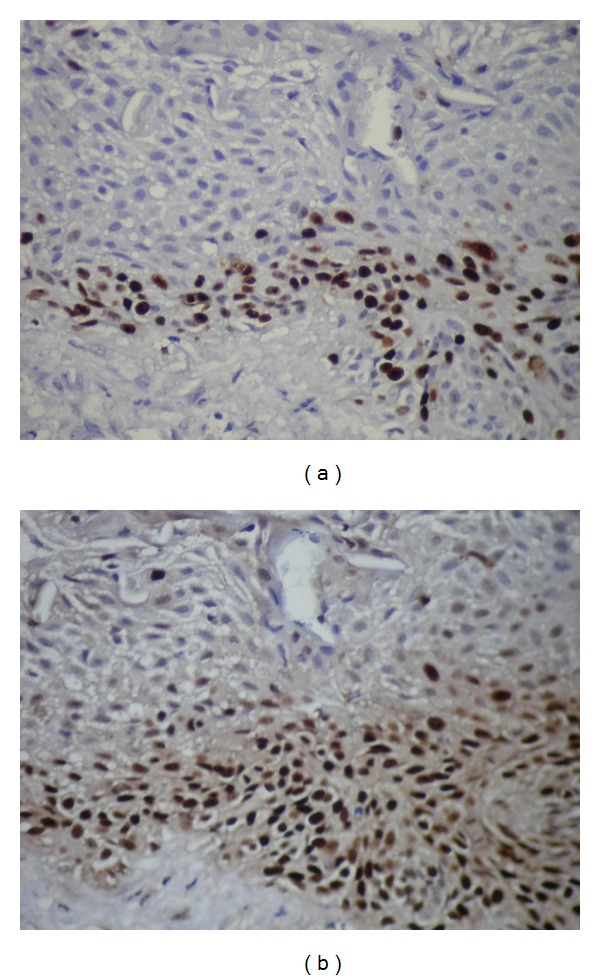
(a) The expressions of Ki-67 in epithelium of keratocystic odontogenic tumors (Ki-67 ×40). (b) The expressions of MCM-2 in epithelium of keratocystic odontogenic tumors (MCM-2 ×40).

Mean Ki-67 and MCM-2 expressions in all specimens are shown in Table 2. The mean Ki-67 and MCM-2 expressions were found 9,64  ±  5,99 and 6,34  ±  3,81 in DF (Figures 6(a) and 6(b)), 11,85  ±  9,01, and 13,6  ±  9,94 in odontogenic cysts, respectively. The mean Ki-67 and MCM-2 expressions in odontogenic cysts were 12,17  ±  4,49 and 19,17  ±  3,76 in RCs (Figures 7(a) and 7(b)), 7,43  ±  3,99 and 7  ±  4,25 in DCs (Figures 8(a) and 8(b)), and 16  ±  13,46 and 15,43  ±  14,04 in KCOTs (Figures 9(a) and 9(b)), respectively. While the mean Ki-67 expressions were statistically significant in DFs, RCS and KCOTs (*P* < 0.01), MCM-2 were significant in RCS and KCOTs (*P* < 0.01). Ki-67 and MCM-2 expressions in RCs and KCOTs were significantly higher than those of DF and DCs (*P* < 0.01). MCM-2 expresions in RCs were statistically significant than KCOTs (*P* < 0.01). 

A significant difference was found between age and mucous cell prosoplasia with calcification (*P* =  0,005 and *P* =  0,024). Ki-67 and MCM-2 expressions were detected in DFs such as 5,82  ±  7,25, and 3,91  ±  4,96 in mucous cell prosoplasia, 11,39   ±  5,82 and 7,5  ±  3,34 in squamous epithelium and 6,5  ±  4,73 and 6,07  ±  3,75 in glandular epithelium respectively (Table 3). Ki-67 expressions were found to be significantly higher in mucous cell prosoplasia (*P* >  0,05), squamous epithelium (*P* =  0,0001), and glandular epithelium (*P* =  0,021) than MCM-2 expressions. Ki-67 and MCM-2 expressions were 8,93 ± 5,90 and 5,58 ± 3,32 in calcifications but no statistically significant difference was observed (*P* > 0,05). MCM-2 expressions were more significant than Ki-67 in DFs with inflammatory cells (*P* =  0,006). 

## 4. Discussion

Cellular proliferation markers including Ki-67, PCNA, AgNOR, and p53 have been used previously to indicate biologic behavior of odontogenic cysts and tumors [1, 5, 8, 10]. The expression of Ki-67 is most likely modulated by morphologic characteristics of epithelial components as well as inflammatory changes [17]. The proliferating cells in DFs showing squamous metaplasia indicated that this change is not a normal change due to advanced age, but a real metaplasia. It is also reported that inflammation might also be effective in squamous changes [9, 17]. In the present study, the expressions of Ki-67 and MCM-2 were 9,64  ±  5,99 and 6,34  ±  3,81 in DFs respectively (*P* < 0,05). These results show that epithelial cells of asymptomatic DFs could be actively proliferating [9]. 47,4% of the DFs, with squamous metaplasia showed inflammatory cells, supporting that inflammation may enhance the squamous changes in healthy DFs.

Inflammation has a puzzling effect on the epithelial lining of different origins. The chronic inflammation may cause chronic irritation and may stimulate proliferation of oral epithelial cells. In several pathologic conditions, inflammation results in epithelial hyperplasia and metaplasia such as RC [18]. Although the histogenesis has not completely known, it is believed that the activation and proliferation of malessez epithelial rests and lining epithelium of RC are related to inflammatory processes [19, 20]. It has been showed that the growth factors and cytokines released by the inflammatory infiltrates in KCOTs might be responsible for the greater proliferative activity [19–22]. The high positivity of markers reflects the cell proliferative activity. The Ki-67 labeling index is significantly higher in KCOT than the DC and RC [23, 24]. The positive number was similar to that for oral epithelium, but greater than for DC and RC. In addition, these previous studies have also shown a similiar trend in the KCOTs and ameloblastoma that had higher proliferation indices of Ki-67 and PCNA than in other kinds of odontogenic cysts including the DC and RC [24, 25]. The biological behaviour of the KCOTs that should be considered to be a benign odontogenic tumor rather than merely a cyst, as its proliferation indices were comparable with those of the ameloblastoma and significantly higher than in other kinds of odontogenic cysts [26]. The greater proliferative appears to indicate an epithelial lining with an intrinsic growth potential. This correlates well with the different clinical behavior of these two groups of lesions, and suggests that the more aggressive behavior observed in KCOTs might be due to the higher proliferation rate of its epithelial lining [23, 24]. In the present study, Ki-67 and MCM-2 expressions were 12,17  ±  4,49 and 19,17  ±  3,76 in RCs, 7,43  ±  3,99 and 7 ± 4,25 in DCs, and 16  ±  13,46 and 15,43  ±  14,04 in KCOTs. Proliferation rates of KCOTs and RCs were higher than the DCs confirming Kim et al. study [23]. In addition, the higher MCM-2 expressions in RC than the KCOTs might be related to the inflammation. DFs with marked inflammatory changes had a higher Ki-67 expression than without marked inflammation and suggested that there might be a direct relationship between the severity of inflammation and proliferation [17]. Several studies have found a direct influence of the inflammation on epithelial cells, either through direct adhesion of the inflammatory cells [27] or through an indirect response to a series of chemokines produced by inflammatory cells [18]. Developmental odontogenic cysts, however, also display this change in the presence of an inflammatory stimulus, as the result of infection or trauma [24]. Li observed that epidermal growth factor expression by odontogenic rests and cysts (KCOT and DC) is related to the presence of inflammation within the adjacent connective tissue. Replacement of the classic parakeratinized lining of KCOTs with nonkeratinizing squamous epithelium has been reported in cases with inflammation present in the cyst wall, and in cases following decompression treatment where inflammation is always present [21]. Inflammation in the connective tissue wall of KCOTs has been found in almost 75% of the cases reported in the literature [21, 24]. Kaplan and Hirshberg reported more frequent (90%) metaplastic squamous epithelium with high inflammatory scores than in cases with low scores (44%) [18]. These findings indicate that there is an association between inflammation and morphology of the epithelial lining in KCOTs. In the present, study inflammation was observed 100% of the RC, 43% of the DC, and 43% of the KCOT.

It is possible that inflammation may alter not only the morphology but also the proliferative potential of the epithelial lining. The morphologic alterations in the epithelial lining of KCOT in the presence of inflammation may also be associated with changes in the proliferative potential, thus, it is reasonable to sugest that growth factors and cytokines released by the inflammatory infiltrate present in the fibrous tissue capsule of KCOTs may be responsible for greater proliferative activity in inflamed lesions compared to noninflamed lesions [22]. However, it is also reported that the inflammation adjacent to the epithelial cyst lining had a localized effect, which induced a focal increase in expression of Ki-67, but not of PCNA. This focal increase did not alter the overall average expression of proliferation markers in the cyst, regardless of inflammation. This can be partially explained by the fact that occasionally the transition to metaplastic epithelium was associated with a decrease in labelling index rather than an increase [18]. In addition to KCOTs, it has been reported that Ki-67 expressions were higher with intense inflammatory cell infiltration were significantly higher than those in RCs with less inflammatory reactions. Proliferative activity in lining epithelium of RCs have different characteristics from those in normal stratified squamous epithelium and that cellular kinetics or turnover of cyst lining epithelium migth be related to the grade of inflammatory changes [28]. In this study expressions of the inflamed specimens were higher for both markers confirming the previous studies that inflamed lesions have greater proliferative activity. Taking into account the role inflammation may play in the stimulation of epithelial proliferative activity and subsequent changes in pericoronal follicle, the evaluation of the proliferation index and apoptosis was performed by Villalba et al. in cases presenting no inflammatory infiltrate. So as to assess epithelial activity as a potential source of pathology in the absence of an added stimulus, they showed that the percentage of proliferation was low in epithelium and mesenchyme of radiographically normal pathologic follicle and markedly lower when compared with DC [29]. However, Ayoub et al. stated that the surface area and the number of immunopositive cells increased with the severity of inflammation in the connective tissue. This could be explained on the assumption that chronic inflammatory reaction could act as stimulators causing epithelial proliferation [30].

Promising biomarkers for diagnosing and prognose of tissues in body include MCM proteins (MCMs 2–7), which assemble in the prereplication complex and are essential for DNA replication in eukaryotic cells [12–14]. All six proteins are abundant throughout the cell cycle and are broken down rapidly on differentiation and more slowly in quiescence. Antibodies against MCMs detect more cells in tissues than other “proliferation” markers such as Ki-67 [15, 31]. Proliferating cells have high levels of MCM, whereas they are not detected in quiescent, differentiated, or senescent cells. They are also potential useful markers of cell proliferation. Their role in replication elongation is supported by numerous studies, but there is still a knowledge gap in this respect. Mašata et al. show that, despite the predominantly different localization of MCM 2 and replication signals, there is still a small but significant fraction of MCM 2 proteins that colocalize with DNA replication foci during most of S phase [32]. The fluorescence localization of the MCM 2 proteins and DNA replication may thus reflect an active function of MCM 2 proteins associated with the replication foci and partially explain one facet of the “MCM paradox” [31, 32].

In conclusion, the differences between the effects of inflammation on Ki-67 and MCM-2 can be related to different regulatory mechanisms of the two markers. Both proliferative markers were statistically significant in mucos cell prosoplasia and squamous epithelium of DFs where Ki-67 expressions were more significant in glandular epithelium of DF. Our results suggest that the higher MCM-2 expressions in RC than the KCOTs might be related to the inflammation and MCM-2 proteins might be more sensitive to inflammatory changes. Further studies are needed to clarfy the MCM-2 expressions in proliferative cells providing whether it is a reliable index of cellular proliferation.

## Figures and Tables

**Table 1:** The distribution of all specimens according to age, sex and localization of lesions.

	Age	Sex		Localization	
	Mean ± (SD)	Female	Male	Right	Left
Dental follicle	27,26 ± 11,54	50 (%71)	20 (%29)	42 (%60)	28 (%40)
Radiculer cyst	43,3 ± 14,24	3 (%50)	3 (%50)	4 (%67)	2 (%33)
Dentigerous cyst	35,57 ± 11,72	5 (%71)	2 (%29)	4 (%57)	3 (%43)
Keratocytic odontogenic tumor	39 ± 12,69	2 (%29)	5 (%71)	1 (%14)	6 (%86)

**Table 2:** Mean Ki-67 and MCM-2 expressions in all specimens.

	Dental follicle	Radicular cyst	Dentigerous cyst	Keratocytic odontogenic tumor
Ki-67 (%)	9,64 ± 5,99*	12,17 ± 4,49*	7,43 ± 3,99	16 ± 13,46*
MCM-2 (%)	6,34 ± 3,81	19,17 ± 3,76*	7 ± 4,25	15,43 ± 14,04*
*P*	**P** = 0,000	*P* = 0,39	*P* = 0,423	*P* = 0,586

*P < 0.01.

**Table 3:** The mean Ki-67 ve MCM-2 expressions in the components of dental follicle.

Dental follicle components	Ki-67 (%)	MCM-2 (%)
Mucous cell prosoplasia	5,82 ± 7,25	3,91 ± 4,96
	**P** = 0,009	**P** = 0,028
Squamous epithelium	11,39 ± 5,82	7,5 ± 3,34
	**P** = 0,001	**P** = 0,001
Glandular epithelium	6,5 ± 4,73	6,07 ± 3,75
	**P** = 0,021	*P* = 0,548
Calsification	8,93 ± 5,90	5,58 ± 3,32
	*P* = 0,324	*P* = 0,103
Inflammation	11,36 ± 6,17	7,69 ± 3,64
	*P* = 0,065	**P** = 0,006
